# Out-of-hospital cardiac arrest treated with prehospital double sequential external defibrillation during eCPR in refractory VF — a case report

**DOI:** 10.1186/s12245-023-00546-5

**Published:** 2023-10-12

**Authors:** Stephan Katzenschlager, Raphael Heck, Erik Popp, Frank Weilbacher, Markus A. Weigand, Christoph Eisner, Christopher Neuhaus

**Affiliations:** Heidelberg University, Medical Faculty Heidelberg, Department of Anesthesiology, Im Neuenheimer Feld 420, Heidelberg, 69120 Germany

**Keywords:** Resuscitation, Extracorporeal cardiopulmonary resuscitation, Double sequential external defibrillation, Advanced life support, Case report

## Abstract

**Background:**

Double sequential external defibrillation (DSED) has demonstrated increased survival with good neurological outcome in a recent randomized controlled trial. DSED has not been studied in patients with extracorporeal cardiopulmonary resuscitation (eCPR).

**Case:**

We present the first case of prehospital eCPR with ongoing refractory ventricular fibrillation (VF), terminated by DSED. After six shocks, return of spontaneous circulation was initially achieved; however, the patient went into recurrent VF. ECPR was performed prehospital, with VF still refractory after three more shocks. DSED successfully terminated VF and showed a further increase in etCO_2_ and near-infrared spectroscopy cerebral oximetry values.

**Conclusion:**

DSED can be a sufficient strategy for patients in refractory VF while on eCPR and should be evaluated in further studies.

## Background

Out-of-hospital cardiac arrest (OHCA) affects about 60/100,000 persons in Europe/year, with 20% having an initial shockable rhythm [1]. Initial shockable rhythm is significantly associated with return of spontaneous circulation and survival to hospital discharge with good neurological outcome [2, 3].

Refractory ventricular fibrillation (VF) is defined by current guidelines as ongoing VF after three defibrillations [4]. Patients with refractory VF represent a typical cohort included in recent extracorporeal cardiopulmonary resuscitation (eCPR) trials [5]. Recent meta-analyses of randomized controlled trials (RCTs) have found increased survival in selected patients undergoing eCPR [6]. If eCPR is not available the concept of early double sequential external defibrillation (DSED) has demonstrated an increase in termination of VF and survival with good neurological outcome [7].

After an intensive literature search, there is no published report on the prehospital use of DSED during ongoing eCPR in refractory VF. In accordance with the CARE guidelines [8], we present the case of a 63-year-old patient, suffering from OHCA with refractory/recurrent VF. Prehospital eCPR was performed, guided by transesophageal echocardiography. Ongoing refractory VF during eCPR was successfully terminated using DSED.

## Case presentation

**Fig. 1:**
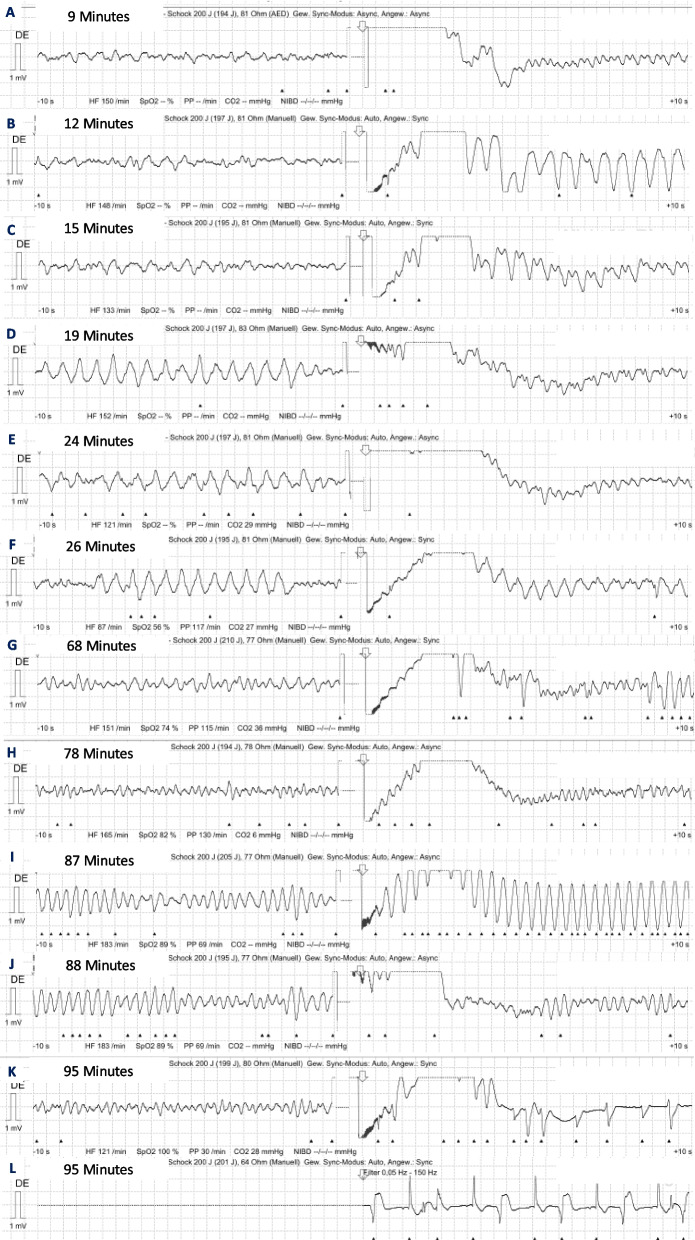
Defibrillations throughout the course of the resuscitation. Defibrillator pads of shocks **A**–**K** were placed in the standard position, while **L** was placed in the anterior–posterior position. Shocks **K** and **L** were delivered with two devices at the same time. Times are stated as minutes after collapse

A 63-year-old patient, without any pre-existing condition, suffered from OHCA during daytime in an urban environment. His wife alerted the emergency medical services and performed bystander cardiopulmonary resuscitation (CPR). Six minutes after the initial call, the ambulance team consisting of one paramedic, one paramedic in training, and one emergency medical technician arrived on the scene. The first rhythm assessed was VF and the first shock was delivered by the ambulance crew (Fig. 1A). Approximately 15 min after the emergency call, the physician response unit, staffed with an anesthesiologist and two paramedics, arrived on the scene. Advanced life support (ALS) was continued with epinephrine and amiodaron applications according to current ALS guidelines [4]. After the sixth shock, the patient achieved a return of spontaneous circulation (ROSC) for the first time, approximately 29 min after the collapse. Due to ongoing hemodynamic and arrhythmogenic instability, the Medical Intervention Car (MIC), a specialized response vehicle, was alerted 45 min after the initial call, and 2 g magnesium was applied. The MIC is staffed with two anesthesia consultants and one anesthesia resident with special training in prehospital emergency medicine and equipped with a primed ECMO machine (Cardiohelp, Getinge Germany, Rastatt, Germany), portable transesophageal echocardiography (TEE), and near-infrared spectroscopy (NIRS).

**Fig. 2:**
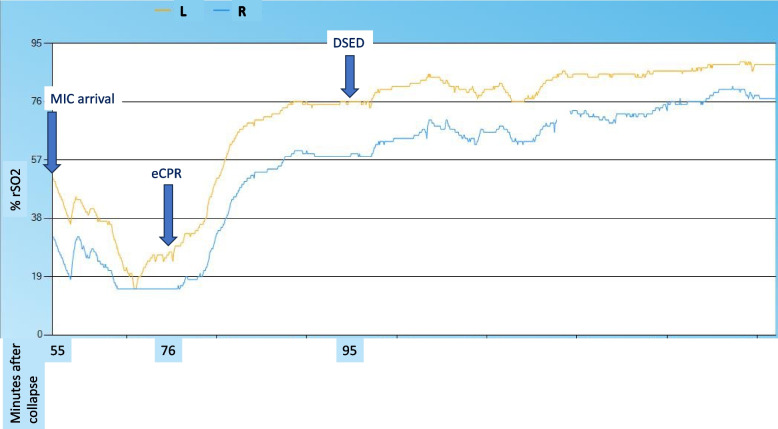
Near-infrared spectroscopy cerebral oximetry values from the MIC arrival until the handover at the cath lab with minutes after collapse at the respective timepoints. Abbreviations: MIC, Medical Intervention Car; eCPR, extracorporeal cardiopulmonary resuscitation; DSED, double sequential external defibrillation

At the time the MIC arrived, the endotracheally intubated patient had ROSC with continuous non-invasive blood pressure (cNIBP) of 83/66 mmHg, end-tidal CO_2_ (etCO_2_) of 40 mmHg, arterial oxygen saturation (SpO_2_) of 92%, and left and right forehead NIRS baseline values of 51% and 32%, respectively (Fig. 2).

As a routine intervention for unstable patients, the placement of two 4-French introducer sheaths in the femoral artery and vein was performed. During this intervention, the patient deteriorated again into recurrent VF, ALS was resumed, and a third dose of 150 mg amiodaron was given, which accounted for a total of 600 mg amiodaron. A team-based decision was made to initiate eCPR by exchanging the two introducer sheaths for ECMO cannulas and connect them to the primed Cardiohelp ECMO. Manual chest compressions were resumed, as no mechanical CPR device was at the patient’s bedside. Confirmation of correct guidewire and cannula position was performed using TEE. During eCPR cannulation, a seventh shock was delivered with the patient remaining in VF.

Return of circulation (ROC) with ECMO flow of 4 l/min was achieved 76 min after the initial collapse, with 47 min of total low flow time. Initial sweep gas flow was 1 l/min with FiO_2_ at 50%, as per institutional guidelines. Due to logistic reasons, prehospital blood gas analysis was not available for this case.

When the patient did not spontaneously convert into a perfusing rhythm on eCPR, 100 mg lidocaine and a bolus of 50 µg norepinephrine were applied. Three shocks were delivered 2, 3, and 10 min after ROC (Fig. 1H–J), respectively. TEE showed increased spontaneous contrast with incipient stasis in the left atrium and ventricle. The decision was made to perform DSED as a measure of last resort. Both shocks were delivered simultaneously by the same person using two identical devices (Corpuls3T, GS Elektromedizinische Geräte G. Stemple, Kaufering, Germany) with ± 200 Joules each. The patient immediately converted into a perfusing rhythm (Fig. 1K, L), with a raise in etCO_2_ from 8 to 39 mmHg and a further increase of NIRS values (Fig. 2). Despite the help of the local fire brigade, extrication from the first floor took 25 min. The patient was transported directly to the catheterization laboratory of the nearest cardiac arrest center where the team arrived 52 min after ECMO flow had been established. The occluded coronary artery (left anterior descending) was successfully stented approximately 200 min after the initial collapse. Unfortunately, the patient died due to a traumatic subarachnoid hemorrhage suffered from the initial collapse and probable aggravation by heparin and antiplatelet therapy.

## Discussion

Despite the poor outcome, we report the — to our knowledge — first case of DSED during eCPR in the prehospital setting. Standard defibrillation was initially successful after the sixth shock, whereas it remained unsuccessful for three shocks during re-arrest. Clinical monitoring such as etCO_2_, NIRS, and TEE showed the impact of rhythm conversion on ECMO. After eCPR initiation, there was an adequate increase in NIRS values; however, etCO_2_ remained low. DSED successfully terminated VF and an increase in etCO_2_ was observed. TEE played a vital role in the decision to perform DSED. Although NIRS showed just below normal values while the patient was in VF, this also increased after the conversion from ROC to ROSC.

According to our protocol, defibrillation, and administration of ALS drugs are withheld during eCPR cannulation. A case-by-case decision can be made by the person cannulating to defibrillate while pausing cannulation.

Despite current guidelines stating that an alternative defibrillation pad position (e.g., anterior–posterior) should be considered for refractory VF [4], this possibility was not considered by the treating prehospital physician or the EMS team.

Early transportation for percutaneous coronary intervention (PCI) under ongoing mechanical CPR (mCPR) can be considered in selected patients. This possibility must be weighed against impaired CPR quality during difficult extrication and transport, as well as the possibility of mCPR-associated injuries. Furthermore, performing PCI during ongoing mCPR can be challenging. To restore brain perfusion as soon as possible, prehospital eCPR is currently believed to have a time and qualitative advantage [9]. In this case, (earlier) transportation under mCPR would have led to an impaired CPR quality with unknown blood flow during the extrication time and transportation to the hospital. Cerebral and coronary perfusion are essential for achieving a favorable neurological outcome. This cannot be guaranteed during challenging extrications, as monitoring the position of the mCPR device requires additional measures, such as invasive arterial blood pressure or TEE. Additionally, CPR-related injuries can occur during transportation over stairs due to misplacement of the mCPR device.

Retrospective observational trials found no significant difference in survival for patients receiving DSED [10, 11]. In contrast, the randomized controlled trial by Cheskes et al. found an increased relative risk for survival with good neurological outcome in patients receiving DSED compared to standard defibrillation (2.21 [95% confidence interval 1.33 to 3.67]) [7]. In this RCT, DSED was performed after the third shock, in contrast with this case, where DSED was used as a last resort. However, none of these studies reported the use of DSED in eCPR patients. Three RCTs on eCPR for OHCA reported a mean range of four to nine prehospital defibrillations [5, 12, 13]; making these patients eligible for DSED. As all cannulations were performed in-hospital, PCI was immediately available and therefore a conversion into a perfusing rhythm could be achieved by opening the occluded coronary artery. In a retrospective cohort study assessing the outcome in patients undergoing out-of-hospital eCPR due to refractory OHCA, a median of two defibrillations is reported, concluding that DSED has not been used [14]. Achieving adequate perfusion before defibrillation is associated with an increased chance of success [15]. A standardized approach for patients on eCPR who remain in VF has not been established. Our institutional guidelines recommend delaying defibrillation for at least 2 min after full ECMO flow has been established. In addition, a vasopressor bolus can be given.

While the ERC ALS guidelines only recommend DSED in research settings [4], ILCOR is currently recommending DSED or vector change in refractory VF [16]. This strategy can also be applied to refractory VF patients with eCPR. This is of special interest for systems where prehospital eCPR is performed, as some patients could benefit from DSED before eCPR is initiated. Additional training should be undertaken before applying this method.

## Data Availability

All relevant data is presented within the manuscript. Further inquiries can be made by addressing the corresponding author Stephan Katzenschlager stephan.katzenschlager@med.uni-heidelberg.de.

## Abbreviations

ALS: Advanced life support
cNIBP: Continuous non-invasive blood pressure
DSED: Double sequential external defibrillation
ECMO: Extracorporeal membrane oxygenation
eCPR: Extracorporeal cardiopulmonary resuscitation
ERC: European Resuscitation Council
ILCOR: International Liaison Committee on Resuscitation
mCPR: Mechanical CPR
MIC: Medical Intervention Car
NIRS: Near-infrared spectroscopy
OHCA: Out-of-hospital cardiac arrest
PCI: Percutaneous coronary intervention
ROC: Return of circulation
ROSC: Return of spontaneous circulation
TEE: Transesophageal echocardiography
VF: Ventricular fibrillation
